# Follicular non-Hodgkin lymphoma recurrence in an uncommon site: the lacrimal caruncle

**DOI:** 10.1093/jscr/rjaf725

**Published:** 2025-09-13

**Authors:** Emirhan Ozkul, Mehmet Serhat Mangan, Ibrahım Bulent Buttanri, Seda Mazmanoglu Atilman

**Affiliations:** University of Health Sciences Haydarpasa Numune Education and Research Hospital, Sadik Eratik Eye Institute, Istanbul, Turkey; University of Health Sciences Sancaktepe Prof. Dr. Ilhan Varank Education and Research Hospital, Department of Ophthalmology, Istanbul, Turkey; University of Health Sciences Haydarpasa Numune Education and Research Hospital, Sadik Eratik Eye Institute, Istanbul, Turkey; Department of Ophthalmology, Acibadem Health Group, Turkey; Department of Pathology, University of Health Sciences Haydarpasa Numune Education and Research Hospital, Istanbul, Turkey

**Keywords:** recurrent follicular lymphoma, caruncular lymphoma, ocular adnexal lymphoma, extranodal lymphoma, rare ophthalmologic malignancy, caruncle

## Abstract

Recurrent follicular lymphoma in the caruncle is an exceedingly rare clinical presentation, with limited cases documented in the literature. This case describes a 58-year-old male with a history of follicular non-Hodgkin lymphoma localized solely to the left caruncle, treated with systemic R-Bendamustine chemotherapy and achieving complete remission. Two years later, the patient presented with a recurrent lesion in the same anatomical site. Clinical and radiological evaluations confirmed the recurrence, and an incisional biopsy was performed, followed by histopathological analysis that reaffirmed the diagnosis of follicular lymphoma. The patient subsequently received 16 sessions of radiotherapy. This case emphasizes the unique challenges associated with managing recurrent caruncular lymphomas, highlighting the critical role of long-term surveillance and a multidisciplinary approach. The rarity of this presentation underscores the need for further research into the pathophysiology and optimal treatment strategies for follicular lymphoma in extranodal sites such as the caruncle.

## Introduction

Follicular lymphoma, a subtype of non-Hodgkin lymphoma, is typically characterized by its indolent nature and frequent extranodal involvement. While primary ocular adnexal lymphomas represent only a small percentage of all non-Hodgkin lymphomas, those originating in the caruncle are exceedingly rare, accounting for <1% of reported cases in the literature [1, 2, 3]. The caruncle, a unique anatomical structure containing conjunctival and cutaneous components, serves as an uncommon site for both benign and malignant neoplasms [4, 5].

In the available literature, there is a sporadic report of follicular lymphoma localized to the caruncle [6], but the recurrence of such a lesion in the same anatomical site has not been documented to date. This case highlights the unique presentation of a recurrent follicular lymphoma in the caruncle after achieving complete remission following systemic chemotherapy. Such a recurrence poses challenges in clinical management and emphasizes the importance of long-term surveillance in patients with ocular adnexal lymphomas. By presenting this case, we aim to contribute to the limited body of knowledge surrounding primary and recurrent caruncular lymphomas and their implications for diagnosis, treatment, and prognosis.

## Case presentation

**Figure 1:**
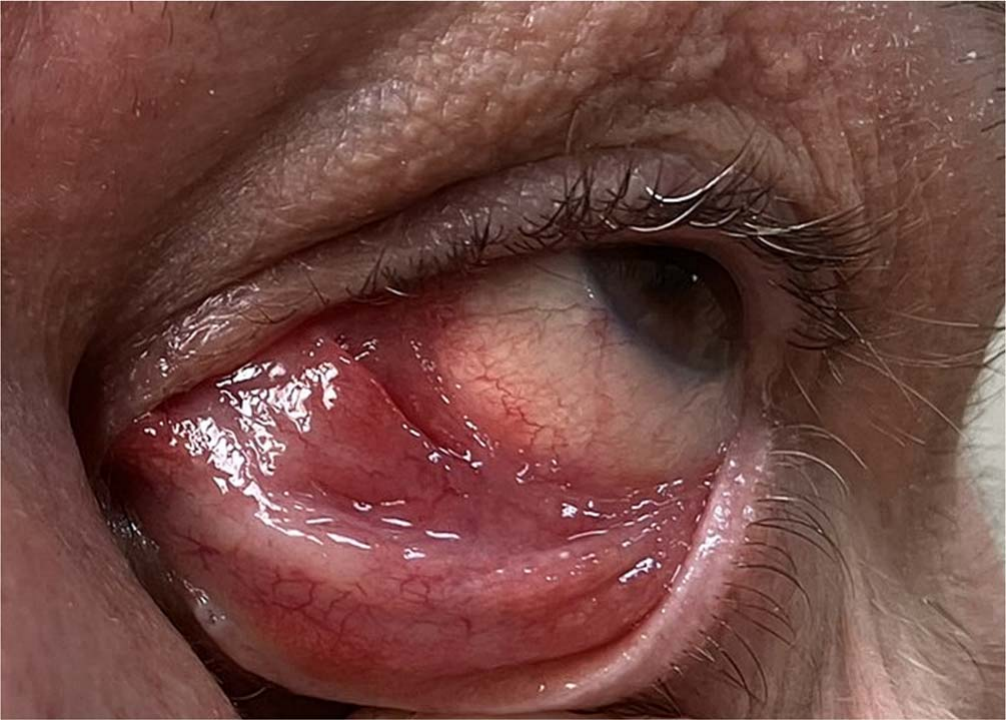
A well-defined pink-red mass in the left caruncular region, appearing localized without ulceration or bleeding. On palpation, the lesion was firm-elastic, with no signs of infection or inflammation in the surrounding conjunctival or adnexal tissues.

A 58-year-old male patient was referred to our ocular oncology unit due to a mass in the left caruncular region. On clinical examination, the best-corrected visual acuity was 20/20 bilaterally, intraocular pressures were within normal limits, and findings from the anterior and posterior segment evaluations were unremarkable. On further examination, a well-defined, pink-red mass was observed in the left caruncular region (Fig. 1) The lesion appeared to be localized without any significant ulceration or bleeding, and it exhibited a firm-elastic consistency on palpation. No signs of infection or inflammation in the surrounding conjunctival or adnexal tissues were noted.

**Figure 2:**
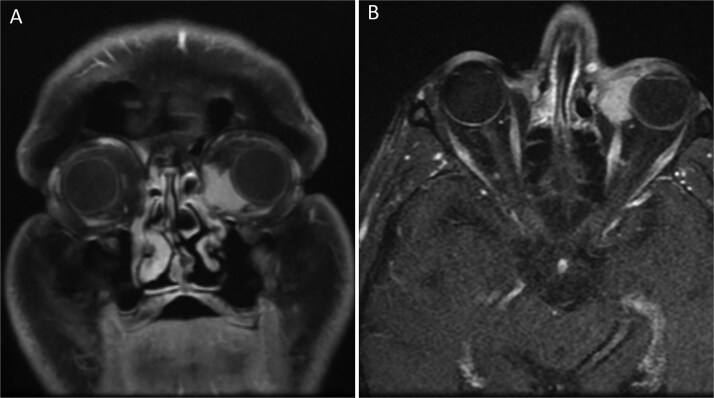
Orbital MRI images of the patient (A) Coronal T1-weighted orbital MRI showing a well-defined lesion in the left caruncular region. The mass measures ~23 × 34 mm and extends to the medial and inferior orbital walls, with homogenous contrast enhancement. No significant infiltration into adjacent structures is observed. (B) Axial T1-weighted orbital MRI showing a homogeneously enhancing mass in the left caruncular region. The lesion extends to the medial and inferior orbital walls without significant involvement of adjacent orbital structures. The mass demonstrates clear boundaries and no evidence of periorbital tissue infiltration.

**Figure 3:**
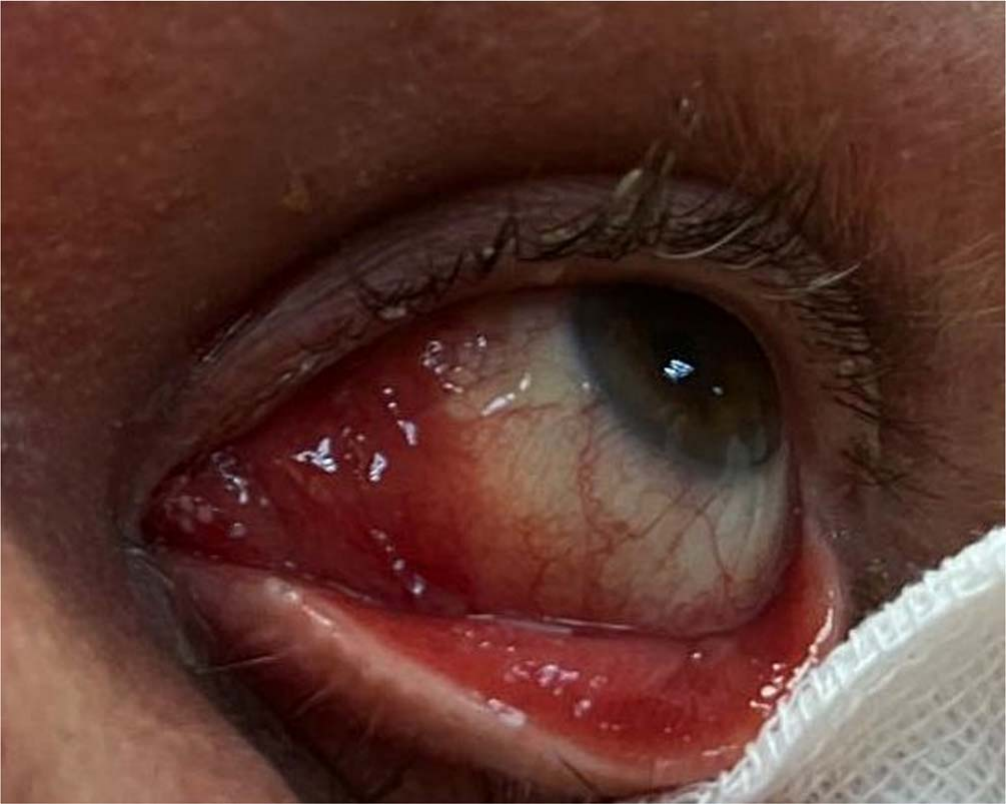
Postoperative clinical image of the left eye showing the caruncular region after incisional biopsy. The surgical site appears clean, with mild erythema and no signs of infection or significant inflammation. The conjunctival and adnexal tissues are intact, and no residual mass is visible.

The patient’s medical history revealed that two years earlier, he had presented with a mass in the left caruncle. Subsequent investigations confirmed a diagnosis of follicular non-Hodgkin lymphoma localized solely to the caruncle. He underwent six cycles of R-Bendamustine chemotherapy. Post-treatment PET-CT scans demonstrated findings consistent with complete remission. Two years later, during a routine follow-up, the patient was found to have a recurrent mass in the same localization. The hematology department referred the patient to the ophthalmology department for further evaluation. Orbital MRI revealed a lesion measuring ~23 × 34 mm at the level of the left medial orbital wall, extending to the inferior wall (Fig. 2A–B), suggestive of orbital lymphoma, with a recommendation for biopsy. An incisional biopsy was performed on the left caruncular mass, and the mass was excised from the caruncle (Fig. 3). Histopathological analysis confirmed a diagnosis of follicular lymphoma. Following the biopsy, the patient received 16 sessions of radiotherapy.

## Discussion

The presented case of recurrent follicular lymphoma in the caruncle represents a unique contribution to the existing body of ophthalmologic oncology literature. While primary follicular lymphoma of the ocular adnexa has been described as a rare entity, its localization and subsequent recurrence in the caruncle are unprecedented. This recurrence highlights the intricate interplay between treatment efficacy and tumor biology [4, 7].

Histopathological findings in this case align with the features of follicular lymphoma, characterized by nodular architecture and the expression of B-cell markers such as CD20 and BCL2 [1, 5]. Previous studies have noted that follicular lymphoma in extranodal sites, including the caruncle, may present with atypical growth patterns due to the unique microenvironment of this location [2, 4].

The management of primary and recurrent caruncular lymphoma involves a multidisciplinary approach. In this case, incisional biopsy followed by radiotherapy was employed, reflecting the standard protocol for localized ocular adnexal lymphomas [3, 7]. However, the recurrent nature of this lesion underscores the need for vigilance in follow-up and possibly adjunctive systemic therapy to prevent further recurrence [5].

Comparison with published cases reveals that caruncular lymphomas often mimic benign lesions clinically, as observed in several reports where initial diagnoses were delayed due to the absence of classic signs such as rapid growth or pigment changes [4, 8]. This case emphasizes the importance of maintaining a high index of suspicion and the role of imaging, such as orbital MRI, in identifying recurrent disease [3].

The rarity of this presentation and its recurrence raise questions about the pathophysiology underlying follicular lymphoma in the caruncle. Potential factors, such as immune privilege of ocular adnexa and local environmental triggers, warrant further investigation [2, 3]. Additionally, this case exemplifies the prognostic challenges posed by recurrent ocular adnexal lymphomas, which often require individualized treatment strategies balancing local control with systemic disease management.

In conclusion, this case not only underscores the unique clinical and pathological characteristics of recurrent caruncular follicular lymphoma but also highlights the critical need for long-term monitoring and multidisciplinary collaboration in managing rare ocular adnexal malignancies. Future studies should focus on identifying risk factors for recurrence and optimizing treatment modalities to improve patient outcomes.

## Data Availability

The data that support the findings of this study are available from the corresponding author upon reasonable request.
